# In Situ Growth, Etching, and Charging of Nanoscale Water Ice Under Fast Electron Irradiation in Environmental TEM

**DOI:** 10.3390/nano15100726

**Published:** 2025-05-12

**Authors:** Hongchen Chu, Qianming An, Xianhui Ye, Duanzheng Wu, Binye Liang, Jiaqi Su, Zian Li

**Affiliations:** School of Physical Science and Technology, Guangxi University, Nanning 530004, China; 2207301016@st.gxu.edu.cn (H.C.); 2307401015@st.gxu.edu.cn (Q.A.); 2207301168@st.gxu.edu.cn (X.Y.); wdz@st.gxu.edu.cn (D.W.); 2207301068@st.gxu.edu.cn (B.L.); 2107401048@st.gxu.edu.cn (J.S.)

**Keywords:** water ice nanostructures, environmental transmission electron microscopy, off-axis electron holography, charging effect

## Abstract

Understanding the formation, structural evolution, and response of water ice at the nanoscale is essential for advancing research in fields such as cryo-electron microscopy and atmospheric science. In this work, we used environmental transmission electron microscopy (ETEM) to investigate the formation of water ice nanostructures and the etching and charging behaviors of ice under fast electron irradiation. These nanostructures were observed to be suspended along the edges of copper grids and supported on few-layer graphene. We varied growth parameters (temperature and time) to produce water ice nanostructures characterized by uniform thickness and enhanced crystallinity. Moreover, we examined the lithographic patterning of water ice at the copper grid edges and its localized etching effects on graphene substrates. Off-axis electron holography experiments further revealed charging phenomena induced by electron beam irradiation, enabling a quantitative assessment of charge accumulation on the ice nanostructures. Our findings demonstrate the controlled growth of ice thin films under high vacuum conditions at cryogenic temperatures, elucidate the etching behavior and charging phenomena of water ice under rapid electron beam irradiation.

## 1. Introduction

Recent research in water ice through environmental scanning or transmission electron microscopy (ESEM or ETEM) has made rapid progress in bridging the gap between fundamental physical processes and practical applications [1,2,3,4,5], from understanding the behavior of ice in natural environments to pioneering innovative nanofabrication techniques [6]. Environmental electron microscopy enables direct, real-time observation of ice nucleation, crystallization, and phase transitions under controlled humidity and temperature conditions [7,8,9]. This helps elucidate the dynamics and mechanisms governing the formation of different ice phases. Investigating how the electron beam interacts with ice is crucial, in particular, the beam-induced effects such as radiolysis, sublimation, and morphological alterations [10,11]. Understanding these interactions leads to improved imaging protocols and low-dose techniques that better preserve the natural state of water ice. Moreover, modern instruments allow for precise control over environmental parameters like temperature, water vapor pressure, and ambient gas composition. These capabilities are essential for simulating natural conditions found in atmospheric, cryobiological, and astrochemical environments, thereby linking laboratory findings to real-world phenomena [12,13,14,15]. Water ice is also increasingly exploited as a resist material in electron beam lithography [16,17,18]. By patterning ice films in both scanning (SEM) and transmission (TEM) modalities, researchers can achieve high-resolution nanofabrication with minimal contamination, opening up new routes for creating nanoscale devices and structures [19,20,21].

Water ice research using environmental electron microscopy faces several significant challenges. Addressing these challenges is crucial for obtaining reliable data and advancing our understanding of water ice behavior in natural and technological contexts. The electron beam can cause radiolysis and the sublimation of water ice, altering its native structure and obscuring the genuine dynamics of phase transitions [22]. Accurately controlling temperature, water vapor pressure, and ambient gas composition is critical yet challenging, as even slight fluctuations can trigger unwanted changes in the ice. Achieving high resolution without inducing damage requires low-dose imaging techniques, which can sometimes compromise signal-to-noise ratios and image clarity. Preparing samples that preserve the intrinsic state of water ice while allowing for in situ observation is difficult. The process must minimize contamination and prevent unwanted crystallization or melting. Despite advances in ETEM, limitations remain in spatial and temporal resolution when trying to capture rapid or subtle changes in the ice structure.

In this study, we employed environmental transmission electron microscopy (ETEM) with an in situ cooling holder and controlled water vapor pipelines to investigate the formation of water ice nanostructures and the etching and charging behaviors of water ice under fast electron irradiation. These nanostructures are suspended along the edges of copper grids and supported on the surfaces of few-layer graphene. We designed a series of experiments to examine the critical growth parameters, namely, temperature and time, required to obtain high-quality ice films with uniform thickness. Furthermore, we explored the lithographic patterning of ice at the edges of copper grids and its localized etching effects on graphene substrates. Notably, off-axis electron holography (EH) experiments revealed the charging effects of ice under electron beam irradiation, and we subsequently quantified the number of charges present on the irradiated ice nanostructures.

## 2. Materials and Methods

Few-layer PELCO graphene products (Ted Pella Inc., Redding, CA, USA) supported on 2000 mesh Cu grids were used as substrates for water ice condensation. The use of the liquid nitrogen cooling cryo-TEM holder (model 636.IN, Gatan Inc., Pleasanton, CA, USA) allows specimen temperature to be changed from room temperature to 95 K. Water vapor deposition was studied using an ETEM (Titan G2 80-300, Thermo Fisher Scientific Inc., Waltham, MA, USA), which was equipped with a high-brightness XFEG electron source, electrostatic biprism, and a Gatan 2k slow-scan CCD camera (Gatan US1000 XP CCD, Gatan Inc.) operated at an accelerating voltage of 300 kV. Electron energy loss spectroscopy (EELS) and off-axis EH were conducted using the microscope. To avoid the suppressive effects of electron beam irradiation on ice growth and deposition, a time-lapse imaging approach was employed, and the illumination dose rate was maintained below $100\;\;e^{-}\;{\overset{˚}{A}}^{-2}\;s^{-1}$ to minimize radiation-induced effects. In this method, the electron beam was blanked (using the Beam Blank function) during the growth period to prevent beam-induced heating and radiolysis. At fixed time intervals, the beam was briefly unblanked to capture snapshots of the ice morphology. To enhance reproducibility, we conducted multiple repetitions and compared the results across different deposition cycles. Prior to each experiment, the liquid nitrogen cryo-holder was baked and plasma-cleaned. And the plasma-cleaned using a model 950 Advanced plasma system (Gatan Inc., USA) to minimize contamination. Prior to inserting the holder into the ETEM, the sample chamber of the ETEM was purged multiple times with high-purity nitrogen gas to ensure cleanliness and eliminate residual gases. Using a normal column vacuum of approximately $2\times 10^{-5}\;\mathrm{Pa}$ and with ambient humidity in the external laboratory environment maintained at approximately 45%, water ice formation was observed when the temperature was reached below approximately 160 K. Repeated experiments confirmed the stability of the actual water vapor pressure within the chamber. Although the exact vapor pressure inside the column could not be directly measured, we assumed it to be equal to the saturated vapor pressure of water at the onset of ice condensation, which indirectly validated its constancy. For detailed formulas, see the Supporting Information. Moreover, the morphology of the deposited ice remained consistent across different cycles, demonstrating the good reproducibility of the growth process under controlled temperature and electron dose rate conditions.

Off-axis EH experiments were performed on a Titan G2 ETEM. To record the holograms, the microscope was operated in the Lorentz mode, with the electrostatic biprism set to 148 V, producing a hologram with fringe spacing of 5.3 pixels. At a magnification of 22.5 k (screen up) and sample rate of 0.42 nm/pixel, each hologram was exposed for 3 s. Elliptical illumination conditions were used to enhance lateral coherence length perpendicular to the fringes. The temperature was maintained at 100 K to facilitate the continuous deposition of ice at the edge of the copper grid. Rod-like ice particles were selected as TEM specimens from randomly selected locations at the edge of the copper grid. The hologram was generated from an original hologram of the rod-shaped ice nanoparticles with 2048-by-2048 pixels by selecting random regions. The object wave was obtained using the spatial phase-shifting method [23], which reconstructs phase images locally, pixel by pixel, from the hologram in real-space.

## 3. Results

### 3.1. Growth of Water Ice at Edges of Cu Grid

**Figure 1:**
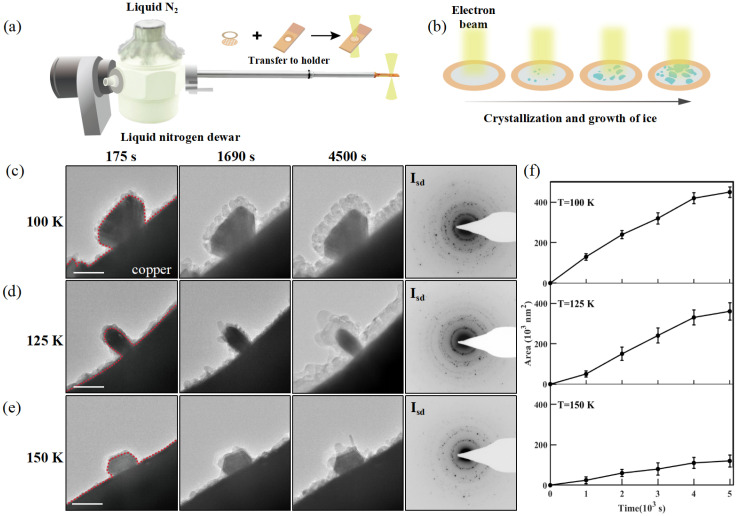
Water ice nanostructures condensed on the edge of the copper grid observed via environmental TEM at cryogenic temperatures. (**a**) In situ cryogenic specimen holder and the copper grids for supporting specimens. (**b**) Schematics for characterizing the condensation and crystallization of ice from water vapor in the TEM vacuum chamber under low electron dose conditions. Representative images of early stages of nucleation and subsequent growth of water ice, with typical SAED patterns showing the mixing of the two phases of ice at (**c**) 100 K, (**d**) 125 K, and (**e**) 150 K. Each image was obtained at fixed time intervals, with a dose rate of $105\;\;e^{-}\;{\overset{˚}{A}}^{-2}\;s^{-1}$ and an irradiation time of 0.5 s. The red dashed line in the figure indicates the boundary between the copper grid and the water ice. (**f**) Size–growth profiles describing the progress of water vapor freezing into nanometer-sized ice crystallites at different temperatures. Scale bars in (**c**–**e**) for TEM images = 500 nm.

To achieve water ice formation within the ETEM, we utilized a liquid nitrogen cooling holder loaded with 5–6 layers of graphene supported on Cu grids, as schematically depicted in Figure 1a. Ice nanoparticles were generated by cooling the graphene substrate to below 150 K under high vacuum conditions (∼$2\times 10^{-5}\;\mathrm{Pa})$ within the ETEM. During this process, residual water vapor present in the ETEM column condensed onto the Cu grids, initiating the nucleation of water ice, as illustrated in Figure 1b.

Since water ice is highly sensitive to electron beam irradiation due to the weak hydrogen-oxygen bonds, it is particularly susceptible to structural damage when exposed to high-energy electrons [10,24]. To minimize radiation-induced damage and preserve the crystallinity of the ice nanoparticles, the electron dose rate was carefully maintained below 100 $e^{-}\;{\overset{˚}{A}}^{-2}\;s^{-1}$, while the total accumulated dose was strictly controlled. We adopted a time-lapse imaging approach to reduce the suppressive effects of the electron beam. The growth morphology was systematically recorded at defined time intervals for three controlled temperature settings. This optimization ensured the structural integrity of the ice crystals throughout the experiment, preventing collapse or dissolution caused by prolonged electron beam exposure.

Figure 1c–e present a series of representative TEM images illustrating the evolution of ice nucleation and subsequent growth at three distinct temperatures: 100 K, 125 K, and 150 K. As the cryogenic holder stabilized at the preset temperature, a thin layer of ice was observed to deposit along the edge of the copper grid at 175 s. The ice continued to grow and expand, exhibiting distinct morphological characteristics depending on the environmental temperature. At 100 K, the deposited water ice formed a dense and uniform layered morphology. The residual water vapor gradually adhered to the surface of the copper grid and accumulated primarily along directions parallel to the substrate, resulting in a relatively smooth, well-ordered, and stepwise-deposited structure without any noticeable rod-like protrusions. In contrast, at 150 K, the growth mode of the ice changed. The structure of the ice crystals grew preferentially in a specific direction, leading to the formation of rod-shaped, elongated structures with pronounced protrusions, as clearly observed in the TEM images. These protrusions were much more pronounced compared to the structures observed at lower temperatures. At the intermediate temperature of 125 K, the ice crystals showed characteristics from both 100 K (a smooth, layered structure) and 150 K (elongated structures). These observations highlight the temperature-dependent nature of ice nucleation and growth dynamics under cryogenic conditions. We adopt the SDAK model to explain why the attachment rate of water molecules on ice crystal surfaces exhibits strong dependence on both crystallographic orientation and temperature [25,26,27]; see Supplementary Information. Specifically, prism edges have high curvature and low local nucleation barriers, which allows water molecules to attach quickly. In contrast, basal facets have higher nucleation energy, which makes it harder for water molecules to attach. Therefore, we propose that temperature influences the molecular attachment coefficient by simultaneously modulating both the surface supersaturation and the nucleation barrier, thereby governing the anisotropic growth behavior of ice crystals across different temperature regimes. As a result, ice crystals preferentially grow along the prism axis, leading to the formation of elongated, rod-like structures. This anisotropic growth behavior reflects the synergistic regulation of molecular attachment kinetics by both temperature and interfacial curvature, further highlighting the strong temperature sensitivity of the ice crystal growth process.

The crystal structure of the condensed water ice was analyzed using selected area electron diffraction (SAED) patterns acquired from micrometer-scale regions at each temperature. The far-right panels in Figure 1c present representative SAED patterns displaying characteristic ring features, indicative of the polycrystalline nature of the deposited ice. These diffraction rings can be indexed to stacking-disordered ice ($I_{\mathrm{sd}}$), which consists of a mixed structure of cubic ice ($I_{c}$) and hexagonal ($I_{h}$) ice phases. Quantitative analysis of the ice growth profiles within the temperature range of 100 K to 150 K revealed a characteristic three-stage kinetic behavior: an initial slow nucleation phase, followed by rapid ice growth, and concluding with a stabilization phase, as shown in Figure 1f. The collected growth profiles confirm that the ice crystals predominantly form within the nanometer size range. The data points in Figure 1f were collected from the same sample area, with each temperature measurement taken from adjacent copper grids within that region. To minimize the impact of electron beam irradiation on the experiment, we employed an indirect recording method and ensured that the electron dose remained consistent during each image capture.

### 3.2. Determination of Cubic Phase of Water Ice

**Figure 2:**
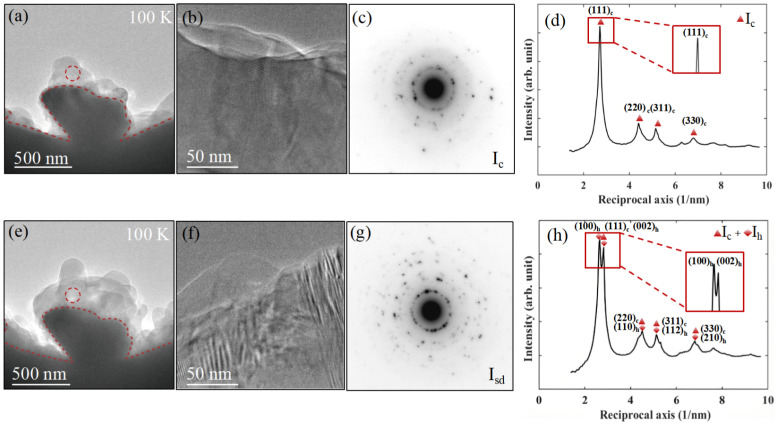
Polymorphic form of ice deposited from water vapor at about 100 K. (**a**–**d**) Low-magnification image, high-resolution TEM (HRTEM) lattice image, corresponding electron diffraction pattern, and the diffraction intensity profile of deposits from randomly selected areas at embryonic stages, showing crystal structure of cubic ice of vapor deposition on the edge of the copper grid. The skewed, diffuse Bragg peaks of the initial crystalline phase indicate the preferential crystallization nucleation of cubic ice. (**e**–**h**) represent (**a**–**d**) after a certain period of growth. The broadening and splitting of the diffraction peaks demonstrate the mixing of cubic ($I_{c}$) and hexagonal ($I_{h}$) phases, which increase gradually with deposition time. The skewed and diffuse Bragg peaks further confirm the unmistakable contribution of hexagonal phase ice. The red dashed line in (**a**,**e**) marks the boundary between the copper grid and the water ice. The red dashed circles in the images indicate the regions from which the SAED patterns in (**c**,**g**) were acquired.

The evolution of the crystal structure of water ice over different growth durations at 100 K was characterized using SAED patterns. Figure 2a presents a representative bright-field TEM image, and Figure 2b shows the corresponding high-resolution TEM (HRTEM) lattice image acquired from the edge of the water ice in Figure 2a, indicating the polycrystalline nature of the newly deposited ice nanostructures. The SAED pattern in Figure 2c was obtained from the region marked by the red dashed circle, and the corresponding integrated diffraction profile in Figure 2d further confirms this structural characterization. In Figure 2d, the radial intensity distribution reveals a prominent ${\left\{111\right\}}_{c}$ peak, along with additional peaks at ${\left\{220\right\}}_{c}$, ${\left\{311\right\}}_{c}$, and ${\left\{330\right\}}_{c}$. Notably, the experimental SAED pattern does not display characteristic peaks associated with hexagonal ice, indicating that the initial ice phase consists purely of cubic ice. This suggests that under high vacuum conditions at 100 K, water vapor condenses onto the cold surface to form a thin layer of cubic ice.

As the freezing process progresses, the water ice layers continue to thicken, as shown in Figure 2e. The corresponding HRTEM lattice image in Figure 2f, obtained from the edge of the water ice in Figure 2e, confirms that the growing ice retains its polycrystalline nature. Figure 2g presents the SAED pattern acquired from the red dashed circular region, and the integrated diffraction profile in Figure 2h reveals diffraction peaks corresponding to the hexagonal phase of ice. Notably, the first appearance of clear hexagonal diffraction peaks occurred after approximately 2.5 h of growth. The broadening and splitting of these peaks indicate the coexistence of cubic and hexagonal ice phases, suggesting a structural transformation during the continued growth of the water ice layers, with the hexagonal ice phase progressively exhibiting a competitive advantage during the structural evolution. In contrast, at higher temperatures (125 K and 145 K), we observed stacking-disordered ice ($I_{\mathrm{sd}}$), which is a mixture of cubic and hexagonal phases, immediately during the early stages of ice deposition. These results suggest that temperature plays a significant role in influencing the growth and transformation of the ice crystal structure.

### 3.3. Time-Dependent Ice Thickness Evolution

**Figure 3:**
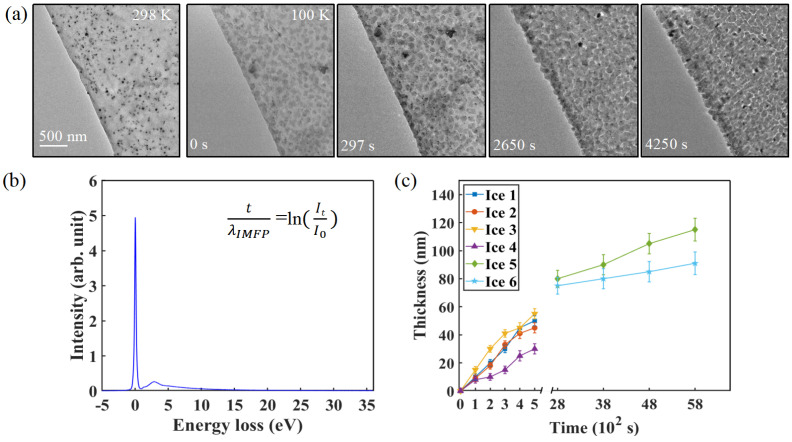
Continuous growth of ice with different thicknesses via in situ cooling ETEM. (**a**) Time-series images illustrating the growth of five representative ice nanocrystals with different thicknesses at the temperature of 100 K. The start time was defined as the moment when the temperature reached 100 K. Each image was obtained at fixed time intervals, with a dose rate of $98\;\;e^{-}\;{\overset{˚}{A}}^{-2}\;s^{-1}$ and an irradiation time of 0.5 s. (**b**) EELS spectra acquired from the area containing ice nanoparticles. (**c**) Thickness increase profiles describing the progress of water vapor freezing to nanometer-sized ice crystallites.

Under the given vacuum conditions (∼$2\times 10^{-5}\;\mathrm{Pa})$ and specimen temperature, residual water vapor in the ETEM column condensed and deposited onto the TEM grids. Figure 3a presents a series of TEM images capturing the cooling process from room temperature to approximately 100 K. Few-layer graphene TEM grids were used as substrates for ice deposition. Notably, small contaminants were observed on the graphene at 295 K, acting as impurity sites for ice nucleation. As the holder temperature reached 100 K, a thin layer of ice nanoparticles deposited onto the graphene surface. With the temperature maintained at 100 K, the ice film continued to grow over time, as shown in the panels of Figure 3a at 297 s, 2650 s, and 4250 s.

To determine the thickness of the deposited ice thin films, we employed EELS using the log-ratio model [28,29,30], as shown in Figure 3b. The corresponding formulas are provided in the Supplementary Materials. To minimize the influence of electron beam irradiation on the measurement of ice film thickness while still acquiring sufficient inelastic scattering signals, the electron beam dose was maintained at $85\;\;e^{-}\;{\overset{˚}{A}}^{-2}\;s^{-1}$.

Figure 3c illustrates the time-dependent thickness evolution of ice films in six selected regions, revealing two stages of water ice growth. In the initial deposition stage, the thickness of the ice nanoparticles increases. In the later freezing stage, however, the growth rate of the ice film thickness decreases significantly, indicating a transition to a steady-state growth regime. And the series of images shown in Figure 3a was obtained from a single cooling cycle during the experiment. A random selection of regions from the sample was observed using a time-lapse imaging method, without continuous electron beam exposure. Additionally, the consistency of the observed experimental phenomena was ensured by comparing the results from multiple repeated experiments, confirming the stability and reproducibility of the findings.

### 3.4. Etching of Ice via Electron Beam Irradiation

**Figure 4:**
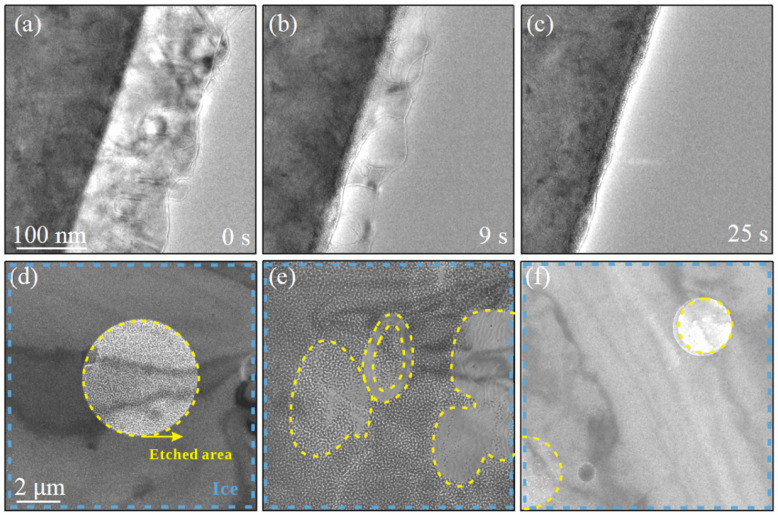
Etching of water ice nanostructures by electron beam irradiation. (**a**–**c**) A series of representative bright-field TEM images showing the etching and sublimation process of ice under electron beam irradiation at 100 K with a total irradiation time of 25 s and a dose rate of $275\;\;e^{-}\;{\overset{˚}{A}}^{-2}\;s^{-1}$. (**d**–**f**) Low-magnification TEM images illustrate the localized sublimation of ice on the graphene substrate within the electron-beam-irradiated region at 100 K. The yellow dashed region marks the area after electron beam etching, while the blue dashed region indicates the ice film substrate. Each image corresponds to an irradiation time of 25–30 s, with a dose rate of $295\;\;e^{-}\;{\overset{˚}{A}}^{-2}\;s^{-1}$.

Figure 4a–c show a series of representative TEM images documenting the lithography process of the ice under intense electron beam irradiation at a dose rate of $275\;\;e^{-}\;{\overset{˚}{A}}^{-2}\;s^{-1}$, with the specimen temperature preset at 100 K. Figure 4a shows a continuous and relatively uniform thin layer of ice distinctly observed along the copper grid edge at 0 s. As the electron beam irradiates the sample for 9 s, the outermost and the edge regions of the ice preferentially begin to sublimate, resulting in noticeable thinning of the ice layer, as shown in Figure 4b. After 25 s of irradiation, the ice completed sublimation, leaving the copper grid edge clearly visible, as shown in Figure 4c.

Figure 4d–f illustrate the etching behavior of water ice deposited on the few-layer graphene substrate. After 25–30 s of irradiation at a dose rate of $295\;\;e^{-}\;{\overset{˚}{A}}^{-2}\;s^{-1}$, we observed localized etching of the ice, which led to the formation of distinct circular voids, as shown in Figure 4d–f, and the corresponding etched patterns in Figure 4e. The yellow dashed region marks the area after electron beam etching, while the blue dashed region indicates the ice film substrate. The clear boundaries of these etched patterns suggest that the etching process of ice can be precisely controlled by adjusting the dose of electron beam and irradiation area under cryogenic conditions.

When a high-energy electron beam interacts with water ice, it transfers energy to the sample, inducing electron-stimulated sublimation. This process primarily involves the displacement of water molecules from the solid phase to the gas phase, leading to the volatilization of ice. The rate of sublimation depends on the electron beam intensity, sample temperature, and irradiation duration. Additionally, high-energy electrons can break molecular bonds within the ice lattice, generating radicals such as hydroxyl and hydrogen radicals, as well as molecular fragments [22]. This bond-breaking mechanism contributes to localized etching at the ice surface, gradually removing material and altering the crystalline structure. As irradiation progresses, surface features such as roughening, pitting, and crater formation may emerge, depending on the irradiation conditions. The accumulation of energy leads to localized structural modifications, resulting in the formation of microscopic pits and changes in surface morphology.

### 3.5. Charging of Ice via Electron Holography

Water ice, as an insulator at low temperatures, accumulates charge when subjected to electron irradiation [31]. Under normal conditions, the electrons cannot easily move within the crystalline lattice, so charge tends to accumulate on the surface. This can lead to a charging effect, where the sample surface becomes negatively or positively charged depending on the relative rate of electron influx and surface electron emission [32]. This effect is especially prominent at low temperatures where the thermal motion of charge carriers is minimal.

**Figure 5:**
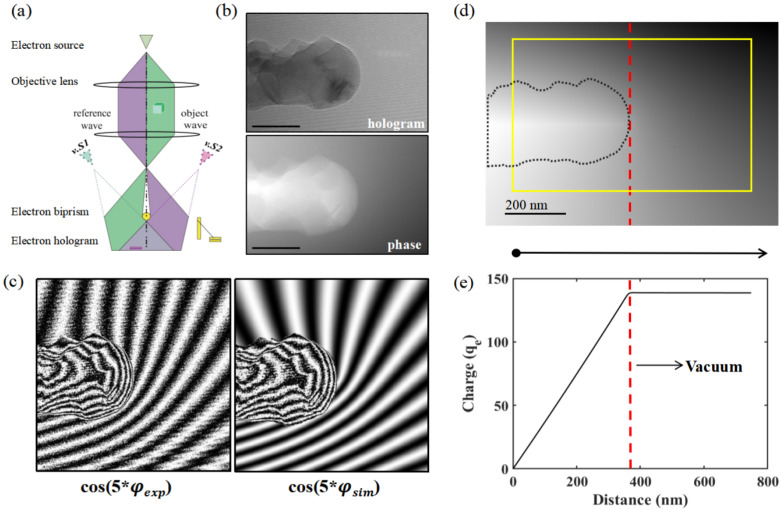
Observation of charging of water ice nanostructures in ETEM. (**a**) Schematics of off-axis electron holography setup in ETEM. (**b**) Experimental hologram and reconstructed phase map. (**c**) Amplified equal-phase contour map $\cos(5\times \varphi_{exp})$ of the reconstructed phase map of a charged ice nanostructure (left) and the simulated phase map contour (right). (**d**) Simulated phase map of a charged needle-shape nanostructure. (**e**) Estimated charge accumulation by the phase gradient integration method. See main text for the details.

We employed off-axis electron holography to investigate the local charging effects in ice under electron beam irradiation [33]. As schematically illustrated in Figure 5a, an electron hologram is formed by the interference of a coherent electron wave transmitted through the sample with a reference wave propagating through the vacuum. This technique enables the measurement of the phase difference (Δϕ) across the sample with a nanometer resolution.

Here, we acquired holograms of a rod-like ice nanostructure suspended at the edge of a Cu grid at 100 K and reconstructed the corresponding phase image, as shown in Figure 5b. The phase map (lower panel in Figure 5b) reveals local variations in the thickness of the ice nanostructure. A closer inspection of the phase map shows a non-flat phase region outside the ice nanostructure, which is further visualized in the experimental phase contour map, $\cos(5\times \varphi_{exp})$, presented in the left panel of Figure 5c. The phase map represents the projected electrostatic potential distribution of the ice nanostructure, while the phase contours reflect the overall distribution of three-dimensionally projected equipotential lines. The presence of extra charges results in the formation of field lines external to the ice nanostructure, whereas the uneven contour distribution within the rod-like ice nanostructure is primarily attributed to its non-uniform thickness.

The external field lines emanating from the rod-like ice exhibit axial asymmetry along its long axis. Such asymmetric field distributions around a charged tip can be attributed to the effect of a perturbed reference wave [34], where the ideal reference wave is altered by interactions with long-range electrostatic or magnetic fields from the sample or its surroundings [35]. For precise phase analysis, it is essential to minimize or effectively eliminate the influence of the perturbed reference wave.

To address this, we adopted the approach proposed by Beleggia et al. [36], employing a linear charge model based on the shape of the charged tip to simulate the surrounding electrostatic fields. The resulting simulated phase image, $\varphi_{sim}$, is shown in Figure 5d. The corresponding simulated phase contour map, $\cos(5\times \varphi_{sim})$, is presented in the right panel of Figure 5c. The details of the charge model and simulation parameters are provided in the Supplementary Materials. By incorporating the effect of the perturbed reference wave, the simulated phase contours $\cos(5\times \varphi_{sim})$ outside the ice nanostructure closely match the experimental contours $\cos(5\times \varphi_{exp})$, as shown in Figure 5c, confirming the validity of the model.

To further quantify the accumulated charge in the ice nanostructure, we performed phase gradient integration on the simulated phase $\varphi_{sim}$. Notably, the simulated $\varphi_{sim}$ is based on a linear charge model, where the phase gradient $\varphi_{sim}$ reflects the electrostatic potential gradient, which is proportional to the projected electric field and indirectly related to the charge distribution. Following the phase gradient integration method proposed by G. Gatel et al. [37], we calculated the phase gradient in the simulated phase map to estimate the total charge present in the ice nanostructure.

The yellow rectangle in Figure 5d marks the integration contour, defining the area used for charge quantification. The integration is performed along the long axis of the rectangle, proceeding from left to right. The resulting accumulated charge distribution is shown by the black line in Figure 5e, which exhibits a linear increase along the length of the rod-like ice. In the vacuum region, where no charge is present, the accumulated charge reaches a saturation level (indicated by the red dashed line). Based on this analysis, the total positive charge accumulated on the ice nanostructure is estimated to be approximately 137 ± 1 $q_{e}$. The influence of the disturbed reference wave can be neglected during the phase gradient integration process for calculating the local charge.

Our EH measurements reveal that the insulating ice nanostructure is positively charged, as shown in Figure 5b. The positive charging of ice under electron irradiation arises from the loss of electrons due to secondary electron emission from the surface, coupled with the absence of a conductive pathway to replenish them. This effect is particularly pronounced in insulating materials such as ice [38,39,40]. The accumulation of net positive charge is further influenced by factors such as ice thickness and electron beam intensity.

## 4. Discussion

### 4.1. The Growth and Etching and Charging of Water Ice Under Fast Electron Irradiation

Our specially designed ETEM provides a controlled environment for studying dynamic processes such as the nucleation and growth of nanoscale water ice under specific gas atmosphere and temperature conditions. This investigation aims to elucidate the mechanisms of ice nucleation and crystal growth. Water vapor molecules condense and form ice nuclei under defined temperature and pressure conditions, after which additional water molecules adsorb onto the growing crystal, facilitating further growth. Our ETEM enables real-time observation of this process, offering insights into ice morphology and crystallography, including the formation of hexagonal ($I_{c}$ or $I_{h}$) ice structures at low temperatures. High-resolution imaging further reveals surface roughness evolution, grain boundary formation, and the development of structural defects during ice crystal growth. Calculations by Malolepsza et al. [41,42] suggest that the transition from cubic ice to a mixed cubic–hexagonal phase may result from dynamic growth conditions. In the early stage of deposition, water molecules preferentially condense near the edge of the copper grid, where strong interfacial interactions favor the formation of cubic ice due to its lower interfacial energy. As the ice layer thickens and moves away from the substrate, interfacial effects decrease. Hexagonal ice becomes thermodynamically favored with lower bulk free energy. Hexagonal ice gradually replaces cubic ice as the dominant phase. Furthermore, the initially formed dense stacking structure of cubic ice provides favorable sites for subsequently deposited water molecules to crystallize into the thermodynamically more stable hexagonal phase, especially in the absence of orientational constraints.

We further investigated the sublimation and etching behavior of ice at cryogenic temperatures. While electron beam lithography typically uses resist polymers [43], controlled ice etching presents a promising approach for cryogenic lithography. This technique could enable high-resolution nano-patterning, surface modification, and sacrificial layer applications that require precise control at low temperatures. The interaction between the electron beam and ice structures may also provide novel strategies for nanofabrication, thin-film deposition, and substrate modification, with potential implications for nanotechnology, semiconductor manufacturing, and materials science.

### 4.2. Water Ice Research with Advanced Electron Microscope and Technologies

Advanced microscopy instrumentation and techniques are crucial for this study. Our ETEM serves as an in situ experimental platform, equipped with a low-temperature sample holder for precise control over temperature and environmental conditions. With spherical aberration correction and a CCD camera, the ETEM enables real-time, high-resolution observation of ice growth dynamics.

Electron diffraction is employed to identify the crystal structure of ice, and the EELS technique facilitates thickness and size measurements [28], allowing for the continuous monitoring of growth evolution. Additionally, electron holography provides quantitative insights into the electrostatic potential and local charge distribution within the ice nanostructure [44]. These complementary techniques collectively enable a comprehensive investigation of water ice growth, sublimation, etching, and surface charging, offering a deeper understanding of nanoscale ice behavior under controlled conditions.

### 4.3. Implication and Future Work

While this study provides valuable insights into the growth behavior of ice under cryogenic conditions in an ETEM setting, a few questions warrant further investigation in future research. First, the influence of external environmental factors on the growth and sublimation behavior of ice requires in-depth exploration. For instance, by precisely controlling the water vapor concentration within the column of an ETEM, the impact of environmental humidity on the growth behavior and crystal structure of ice can be systematically studied. Second, future research could be expanded to include more complex ice systems. For example, by introducing specific gases into the ETEM under cryogenic conditions, the formation mechanisms and properties of doped ice or polycrystalline structures could be studied. This study is also limited by the uneven thickness of the ice samples. Although simulations help minimize the contribution of the mean inner potential to the phase gradient, it is still not possible to fully replicate the true experimental conditions [37]. Future work could focus on optimizing electron holography techniques for uniformly thick ice, improving the measurement accuracy of samples.

## 5. Conclusions

In summary, our TEM investigation explored the controlled growth of ice under cryogenic conditions. In situ TEM experiments at 100 K, 125 K, and 150 K demonstrated a strong temperature dependence in growth behavior, with tightly packed layers at 100 K, rod-like structures at 150 K, and a hybrid mode at 125 K. SAED analysis confirmed the gradual evolution of ice into a cubic ($I_{c}$) and hexagonal ($I_{h}$) phase mixture. Ice growth followed a three-stage process: slow nucleation, rapid growth, and stabilization. Cryogenic TEM at 100 K further revealed that early-stage ice initially formed as pure cubic ice before transitioning into a mixed phase. The thickness evolution, measured via the EELS log-ratio method, showed an initial rapid increase followed by a gradual slowdown, aligning with the observed growth kinetics.

Under high-dose electron beam irradiation, we observed the localized sublimation of ice at the edge of the copper grid and distinct etched patterns on the graphene substrate, demonstrating that electron beam irradiation can precisely control ice sublimation and etching under cryogenic conditions. Additionally, off-axis EH was employed to obtain electron phase images, allowing for the estimation of local charge distribution in rod-shaped ice nanoparticles. Our findings provide insights into the behavior of ice growth, sublimation, and charge distribution under cryogenic conditions. This work offers new perspectives for practical applications in cryogenic material science and nanotechnology, particularly in the development of ice etching techniques and charge-controlled nanostructuring.

## Data Availability

The data presented in this study are available on request from the corresponding author.

## Supplementary Materials

The following supporting information can be downloaded at: https://www.mdpi.com/article/10.3390/nano15100726/s1, Figure S1. Condensation process of ice on graphene observed via in situ cooling TEM. Figure S2. Sublimation process of ice observed via in situ cooling TEM. Figure S3. Visualization of sublimation process of ice on the edge of the copper grid using in-situ cooling TEM. Refs. [25,26,27,28,29,30,36,45] are in the Supplementary Materials.

## Abbreviations

The following abbreviations are used in this manuscript:

TEM	Transmission electron microscopy
SAED	Selected area electron diffraction
HRTEM	High-resolution TEM
ETEM	Environmental transmission electron microscopy
ESEM	Environmental scanning electron microscopy
EH	Electron holography
EELS	Electron energy loss spectroscopy
$I_{c}$	Cubic ice
$I_{h}$	Hexagonal ice
$I_{\mathrm{sd}}$	Stacking-disordered ice
