# Supplementary material for: In Situ Growth, Etching, and Charging of Nanoscale Water Ice Under Fast Electron Irradiation in Environmental TEM
Source: Nanomaterials (Basel). 2025 May 12;15(10):726. doi: 10.3390/nano15100726 (PMC12114155; doi:10.3390/nano15100726)
Supplement: Supplementary file 1 [file nanomaterials-15-00726-s001.zip › nanomaterials-3619395-supplementary.pdf]

Article

Supplementary Materials to: In Situ Growth, Etching, and Charging of Nanoscale Water Ice Under Fast Electron Irradiation in an Environmental TEM

Hongchen Chu, Qianming An, Xianhui Ye, Duanzheng Wu, Binye Liang, Jiaqi Su and Zian Li *

School of Physical Science and Technology, Guangxi University, Nanning 530004, China; 2207301016@st.gxu.edu.cn (H.C.); 2307401015@st.gxu.edu.cn (Q.A.); 2207301168@st.gxu.edu.cn (X.Y.); wdz@st.gxu.edu.cn (D.W.); 2207301068@st.gxu.edu.cn (B.L.); 2107401048@st.gxu.edu.cn (J.S.)
* Correspondence: zianli@gxu.edu.cn

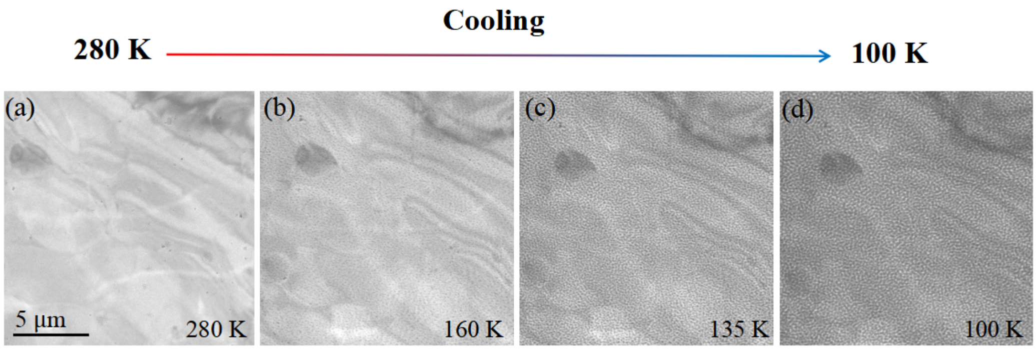

Figure S1. Condensation process of ice on graphene observed via in situ cooling TEM. (a) Initial state at room temperature. (b-d) Representative images of water vapor in the TEM vacuum chamber continuously deposits on the cryogenic graphene as ice with decreasing temperature. Scale bars for TEM images = 0.5 μm.

In addition to observing ice growth at the edge of the copper grid, we observed the temperature-dependent deposition and sublimation processes of ice on both graphene and Cu-grid edges by controlling the temperature. During the cooling process, water vapor in the TEM vacuum chamber condensed onto the cryogenic graphene substrate, forming a uniform ice layer as the temperature decreased from room temperature to 100 K. At 293 K, the graphene-surface was observed to be clean. As the temperature dropped below 160 K, water vapor in the chamber began to condense, and the ice layer gradually thickened. By 100 K, a dense and relatively uniform ice layer had formed, demonstrating the effectiveness of low temperatures in promoting ice nucleation and growth.

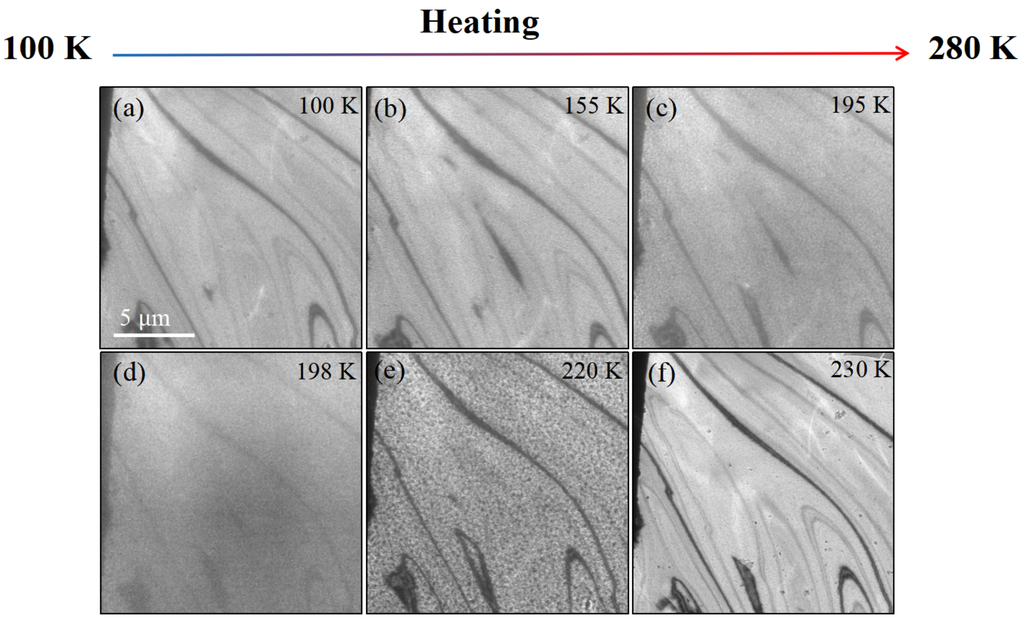

Figure S2. Sublimation process of ice observed via in situ cooling TEM. (a) Initial state at room temperature. (b)-(f) Representative images showing the sublimation of ice on the sample with increasing temperature. Scale bars for TEM images = 0.5 μm.

We conducted in situ heating TEM experiments on the ice on the graphene-substrate, and the sublimation behavior exhibited clear substrate-dependent differences. On the graphene surface, noticeable thermal expansion of the ice was observed. As the temperature increased, sublimation began around 193 K, and the ice layer transitioned into small crystalline ice particles, which rapidly sublimated and disappeared. By 230 K, the graphene surface was once again clean.

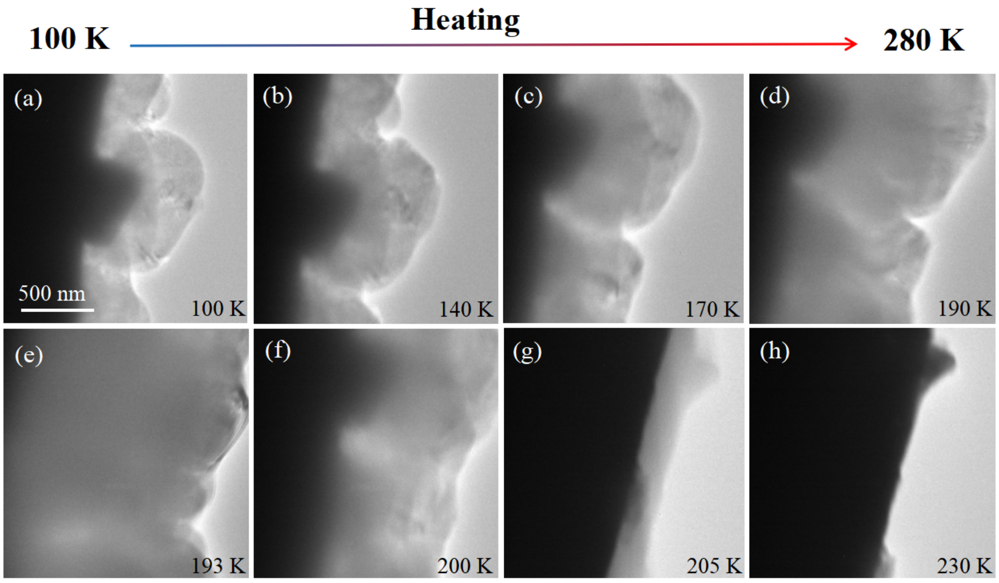

Figure S3. Visualization of sublimation process of ice on the edge of the copper grid using in-situ cooling TEM. (a) Initial state of ice at 100 K. (b-h) Representative images of the sublimation of ice with increasing temperature. Scale bars for TEM images = 500 nm.

We continued the heating experiments on the ice at the edge of the copper grid. The ice at the edge of the copper grid showed a more pronounced and continuous expansion process. With increasing temperature, structural rearrangement of water molecules within the lattice caused further ice expansion. Sublimation commenced at approximately 193 K, with the ice layer thinning progressively until it completely disappeared by 230 K. The pre-sublimation expansion observed on the copper grid highlights the influence of the substrate, where stronger ice-substrate interactions and differences in thermal conductivity modulate the sublimation process.

SDAK model:

Prof. Kenneth G. Libbrecht from the Massachusetts Institute of Technology proposed the Structure-Dependent Attachment Kinetics (SDAK) model[1–3]. According to this model, the molecular attachment coefficient on a given facet of an ice crystal can be expressed as:

$$\alpha_x(\sigma_{\text{surf}}) = A_x e^{-\frac{\sigma_{0,x}}{\sigma_{\text{surf}}}}, \tag{S1}$$

where σ_{surf} is the surface supersaturation, is A_x a pre-exponential factor representing surface diffusion, and $\sigma_{0,x}$ corresponds to a step energy derived from the nucleation barrier on that facet.

The EELS Log-ratio model for measuring ice thickness:

$\frac{t}{\lambda_{IMFP}} = \ln\left(\frac{I_t}{I_0}\right)$ where t is the sample thickness, λ_{IMFP} is the inelastic mean free, I_t is integrated area of the entire EELS spectrum, and I_0 is the integrated area of the zero-loss peak [4]. The λ_{IMFP} value can be estimated using the following equation, $\lambda_{\text{IMFP}} \approx \frac{106FE_0}{E_m \ln(2\beta E_0/E_m)}$, $F = \frac{1+E_0/1022}{(1+E_0/511)^2}$, where E_0 (keV) is the electron energy, β (mrad) is the collection angle of the EELS spectrometer, F is the relativistic factor, and E_m (eV) is the average energy loss for the examined material [5,6]. The value of E_m is typically dependent on the refractive index and composition of the material. It can be calculated using the formula, $E_m = 7.6Z_{\text{eff}}^{0.36}$. Using these experimental formula, we calculated the inelastic mean free path for ice at 300 keV, which was found to be 214 nm. This value was then used in subsequent thickness calculations.

At the same time, we implemented multiple measures during data acquisition and processing, and conducted repeated experiments to verify the reliability and reproducibility of the results, while keeping the errors within an acceptable range to ensure that they do not have a substantial impact on the final conclusions. First, we employed an EELS spectrometer with ultrahigh energy resolution. The energy resolution was calibrated by measuring the full width at half maximum (FWHM) of the zero-loss peak (ZLP), which reached approximately 1 eV. This high resolution ensures a clear separation between the ZLP and the low-loss region, enabling the extraction of accurate thickness information. Before data collection, we performed ZLP alignment to minimize peak drift. In addition, to eliminate the effect of sample drift, we allowed the sample to stabilize at 100 K after cryogenic deposition before collecting the spectra.

Considering the beam sensitivity of ice, we strictly controlled the electron dose during EELS acquisition to minimize beam-induced damage while ensuring sufficient inelastic scattering signal. We optimized the spectrum acquisition parameters and integration range. Specifically, we carefully set the integration window for the zero-loss peak (I_0) to avoid signal leakage or the inclusion of low-loss background. Considering the beam sensitivity of ice, we strictly controlled the electron dose during EELS acquisition to minimize beam-induced damage while ensuring sufficient inelastic scattering signal. We optimized the spectrum acquisition parameters and integration range. Specifically, we carefully set the integration window for the zero-loss peak (I_0) to avoid signal leakage or the inclusion of low-loss background.

Calculation Method for Electron Dose Rate:

The dose rate was determined based on measurements from the ETEM's calibrated fluorescent screen and software readout. The beam size and illumination area were estimated in real space using the calibrated scale displayed on the screen. The total beam current was measured either via the screen current monitor or a Faraday cup when available. The electron dose rate was then calculated using the relation:

$$\text{Dose rate } (\text{e}^- \text{ Å}^{-2} \text{ s}^{-1}) = \frac{I}{e \cdot A}, \tag{S2}$$

where I is the beam current in amperes, e is the elementary charge (1.602×10^{-19} C), and A is the illuminated area in Å^2. This method enabled real-time monitoring and adjustment of the dose rate during imaging to ensure accurate structural observations under low-dose conditions.

Equation for Ice Saturation Vapor Pressure:

we adopted an empirical formula proposed by Murphy et al.[7] to calculate the saturation vapor pressure of ice at low temperatures:

$$P_{\text{ice}} = \exp\left(9.550426 - \frac{5723.265}{T} + 3.53068 \ln(T) - 0.00728332T\right), \tag{S3}$$

where P_{ice} is the saturation vapor pressure of ice, and T is the temperature in Kelvin. This formula enables the estimation of the saturated water vapor pressure at a given temperature.

Theoretical model expression:

$$\varphi(x,y) = \frac{KC_E}{8\pi\epsilon_0}\left\{4Ly + 4xy \arctan\left(\frac{y-L}{x}\right) - 4xy \arctan\left(\frac{y+L}{x}\right) - (L^2 + x^2 - y^2)\log\left(\frac{x^2 + (y-L)^2}{x^2 + (y+L)^2}\right)\right\}, \tag{S4}$$

the shape of the ice is approximated as an ellipsoid with a major semi-axis a = 233 μm and a minor semi-axis b = 127 μm. Once the shape of the ice is determined, the value of K is obtained through a best fit to the phase difference image in the vacuum region around the ice, where K is an appropriate constant with units of surface charge density[8]. The best fit with K = 1.1 e/μm^2 and an interference distance $D = 1\,\mu m$, which shows a relatively good agreement with the experimental data.

1. Libbrecht, K.G. The physics of snow crystals. *Reports on progress in physics* **2005**, *68*, 855.
2. Libbrecht, K.G. Toward a Comprehensive Model of Snow Crystal Growth: 10. On the Molecular Dynamics of Structure Dependent Attachment Kinetics. *arXiv preprint arXiv:2012.12916* **2020**.
3. Persad, A.H.; Ward, C.A. Expressions for the evaporation and condensation coefficients in the Hertz-Knudsen relation. *Chemical reviews* **2016**, *116*, 7727–7767.
4. Egerton, R.F. Electron energy-loss spectroscopy in the TEM. *Reports on Progress in Physics* **2008**, *72*, 016502.
5. Malis, T.; Cheng, S.; Egerton, R. EELS log-ratio technique for specimen-thickness measurement in the TEM. *Journal of electron microscopy technique* **1988**, *8*, 193.
6. Egerton, R.; Cheng, S. Measurement of local thickness by electron energy-loss spectroscopy. *Ultramicroscopy* **1987**, *21*, 231.
7. Murphy, D.M.; Koop, T. Review of the vapour pressures of ice and supercooled water for atmospheric applications. *Quarterly Journal of the Royal Meteorological Society: A journal of the atmospheric sciences, applied meteorology and physical oceanography* **2005**, *131*, 1539.
8. Beleggia, M.; Kasama, T.; Larson, D.J.; Kelly, T.F.; Dunin-Borkowski, R.E.; Pozzi, G. Towards quantitative off-axis electron holographic mapping of the electric field around the tip of a sharp biased metallic needle. *Journal of Applied Physics* **2014**, *116*.
